# Factors affecting consumer attitudes towards using digital media platforms on health knowledge communication: Findings of cognition–affect–conation pattern

**DOI:** 10.3389/fpsyg.2023.1008427

**Published:** 2023-01-26

**Authors:** Siqi Zeng, Xinyi Lin, Liting Zhou

**Affiliations:** School of Urban Culture, South China Normal University, Guangzhou, China

**Keywords:** health knowledge marketing, digital health literacy, digital media, attitude, health communication, CAC pattern

## Abstract

As the world enters the second year of the pandemic, many posts are marketed to promote products under the guise of spreading knowledge to fulfill the users’ requirement for health knowledge. Current research, however, has primarily focused on methods to improve health literacy rather than the impact of this new form. To remedy this deficiency, this research use Cognition–Affect–Conation Pattern (CAC) to investigate digital media users and to determine attitudes towards this new form and influential. Data was collected in December 2021 from 314 users in Guangzhou, China, *via* a Likert-type scale developed by the researchers. Regression analyzes were also performed. Research has shown that consumer attitudes in health knowledge marketing fall within the standard learning hierarchy, with consumer self-cognition and information quality cognitions showing a positive relationship with their affect and conation. At the same time, affect did play a mediating role in this model. The results of our study provide constructive solutions for companies to optimize the communication environment of health spread in society. The findings not only provide researchers with a new perspective to study the impact of interactive digital media communication on health knowledge, but also help identify users’ information needs and formulate effective strategies to increase consumers’ understanding and application of health knowledge and products. Providing health knowledge content and leveraging digital media to develop well-established communication channels is important to foster relationships with customers.

## 1. Introduction

Through the ‘connection’ approach, internet technology is rapidly revolutionizing people’s lives by interconnecting various scenarios of our daily lives such as production, office, education, consumption, etc. The business paradigms of enterprises are constantly being subverted with the advent of the internet and the era of big data, and ‘Internet plus’ has become an inexhaustible driving force for innovation-driven economic development (Dong et al., 2022). As the foundation for enjoying a better life, public health is the core element of sustainable development (World Health Organization, 2022), and it is gradually developing towards ‘Internet + Medical Care’. China’s digital health management market has seen tremendous growth (iiMedia Report, 2021) under the auspices of ‘Healthy China 2030’ and initiative and the impetus of several factors, including population pressure, chronic sickness patterns, and artificial intelligence. After the spreading of COVID-19 (Zhang and Li, 2022), worries about global health transmission in the post-pandemic coexist with internet security issues. People’s attention to health has greatly increased, and the implementation of health transmission has become crucial.

As a product of the development of the internet, digital media provides a communication method for medical institutions or related companies. They can create audiovisual media content disseminating on digital platforms (Twitter, Instagram, WeChat, TikTok, etc.) to disseminate health knowledge (Allegrante and Elaine, 2019; Huo et al., 2019; Petersen et al., 2019; Riley et al., 2019). On the basis of Web2.0, they concentrate on important terms such as ‘individuals’, ‘connection’, and ‘sharing’, ultimately constructing a unified ecosystem of ‘content network’ and ‘relationship network’ (Peng, 2013). Thanks to digital media, health knowledge exchange, especially in the post-epidemic era, can occur instantly without geographical constraints and incorporate innovative elements in diverse ways to capture human attention and foster social connections. Also, the users here may be divided into two groups. One is wherein many users exist as ‘nodes’ all over the internet (Peng, 2016). Most of them are non-professional users and have the right to freely express their health opinions. However, they are often viewed as a source of health knowledge takers or publishers of scattered opinions. The other group concerns individuals who have obtained a certain professional level, such as doctors, hospitals, internet companies of medical industry, and vertical media in medical area, who use WeChat Official Account (WOA), TikTok, Weibo, and other digital platforms to form a communication matrix. By using pictures, texts, short videos and other forms, they transform medical research results into health knowledge, thus improving the health literacy of most users (Jackson, 1992). In the wake of the outbreak, digital media has become a vital source of health information and expertise for people throughout the globe, not just in China (Tennant et al., 2015; Fergie et al., 2016; Househ, 2016).

As China’s biggest medical field connector and professional service provider in the digital sector, DXY.cn, has grown from a website that first offered literature search services to a huge company that can provide a variety of services for medical staff, public users, and corporate users. This background is consistent with the development of internet medicine, so it is typical of the study (Yu et al., 2019; iResearch, 2021). *Inter alia*, DXY.cn possesses a complete media matrix, including Weibo, WOA, TikTok, application, etc. Among them, there are a total of 62 WOA, covering various fields in medicine, furnishing vertical content for users with different needs (Hao, 2019; iiMedia Research, 2020). The research team conducted a study of content posted on one of its WOA, ‘Dr. Dingxiang’. Aside from the purpose of sharing basic health knowledge, articles that revolve around knowledge but aim to promote their products at the end of them are also increasing in recent years. Enterprises would utilize digital media to spread health knowledge to the public as well as to attract potential consumers by richening product value, thus promoting consumerism. For example, links to the product relevant to the contents shared as well as posters for tutorials advertisements to the product can be attached at the end of each article or video sharing certain facts and health knowledge. Thus, when readers recognize that they match some of health issues in the article, they would develop their desire to purchase the relevant products recommended and subsequently act on this desire. This phenomenon is not only seen in Mainland China. Also, in America, the website ‘Health.com’ owned by Dotdash Meredith has also produced articles that featured such product marketing strategies.

Most research underwent consisted of fervent discussion concerning how practitioners or a specific population would use digital media to promote good health practices within certain patient groups, i.e., pregnant women, or to prevent the development of conditions such as diabetes and cancer (Beaudoin and Hong, 2017; Collins et al., 2020; Williams et al., 2021; Álvarez-Pérez et al., 2022; John et al., 2022). Furthermore, some research has concluded that health promotion should be included as a core lesson in schools to tackle fake news which spread at a speed comparable to a virus replicating in the body. With this incorporation, public awareness of basic health and hygiene would be increased (Mandelbaum, 2021). However, less research was found to focus on the distribution of health knowledge from the aspect of enterprises and consumers. Some researchers have conducted research from health communities on digital media, resulting that some aspects can help improve the health information environment, including the relatability of the contents shared with the audience, and the mannerism of digital information which can also reduce social inequality rooted in the information shared in certain health content, thus promoting the development of health improvement strategies (Norman and Skinner, 2006; Tennant et al., 2015; Kanthawala and Peng, 2021; Ye et al., 2022). In the area of healthcare marketing, some researchers have used the theories of KAP and DoI to investigate the knowledge generations of traditional Thai medicine (TTM) and to design the most ideal marketing strategy for the relevant products (Senachai et al., 2022). However, the aim of the research lies within the product but not to share health knowledge.

In conclusion, knowledge marketing has become a common phenomenon in health communication. However, less research has been carried out in this area. As consumers’ attitude of this marketing mode are still unaware, there is significance in conducting research concerning the nature of such marketing strategy in the near future. As such, we would like to propose the following questions regarding the local health communication scenario: (1) Is it effective to share health knowledge through articles? (2) What content would attract the attention of consumers? (3) Will the addition of product recommendations influence health communication? This paper is conducted with Cognition-Affect-Conation Pattern (CAC) as its foundation to investigate the relationship between self-cognition on health knowledge, information recognition, affect and conation.

The study of expression effects is rather new in the field of media effects, and research into the mechanisms is still scarce (Valkenburg et al., 2016). This study fills a gap in quantitative research on this phenomenon in the field of health communication. Understanding consumer attitudes will enable us to better comprehend the role of digital media as a channel for health information in the dissemination of general health knowledge. Last but not least, it will also enable us to identify users’ information needs, which will encourage businesses to optimize their information design on health communication in order to better satisfy users.

## 2. Literature review and hypothesis development

### 2.1. Theoretical background and related studies: Cognition-affect-conation pattern

Attitudes are the tendency of people to evaluate and behave towards something based on their morals and values. Connecting objects and evaluations, it is reaction to items based on a degree of preference. Based on attitude theory, it is feasible to accurately predict and explain a vast array of human behaviors (Fishbein and Ajzen, 1975). The Three-Component Attitude Theory, which combines object and personal elements and provides an overview of the structure of attitudes, was first proposed by Rosenberg and Hovland (1960) and Ajzen and Fishbein (2011). With the application of psychology to attitude theory, the American psychologist Albert Ellis advanced the Affective-Behavioral-Cognitive Model of Attitude. The ABC model describes attitudes as a combination of cognitive, affect and behavioral inclinations (Aronson et al., 2010). However, Ellis’s research was based on the reality that individuals were being disadvantaged and centered on the irrational ideas involved. In response, Fishbein et al. modified this concept as the Cognition-Affect-Conation Pattern (CAC), claiming that a person’s cognition, affect and conation to act are significant components in determining their attitude (Fishbein and Ajzen, 1975). They posit that an individual’s intention and willingness to demonstrate a behavior (i.e., conation) are subjected to his attitude toward that behavior (i.e., affect), which is shaped by his/her belief and perceptions about demonstrating the behavior (i.e., cognitive). The explanation of this model has improved later scholars’ comprehension of the mechanisms of public information processing.

**Figure 1:**
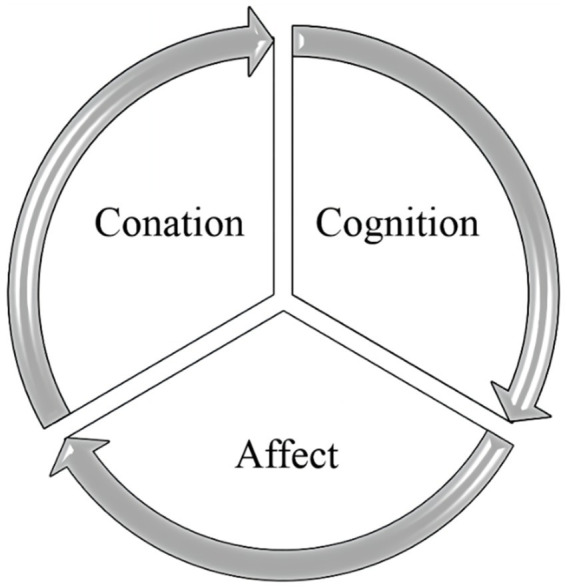
Cognition-affect-conation pattern (CAC) pattern.

In this model (Figure 1), cognition consists of the perception and comprehension of objects, which, when combined, produce beliefs about those things. At its most basic, affect is the subjective experience of liking or hating something, based on one’s intuition and feelings. In other words, affect can have or exhibit positive or negative feelings about the object. These are primarily evaluative as favorable or unfavorable, are a current assessment, considered an essential aspect of their attitude (Kim et al., 2013). Conation is inclination to act on the basis of both current perception and emotional feedback. It is not the action itself, but rather the state of readiness just prior to action (Fazio, 1995; Ajzen and Fishbein, 2011). Positive attitudes tend to be associated with positive tendencies, whilst negative attitudes tend to be associated with avoidance. To explain the different levels of motivation of consumers towards attitude objects, researchers have developed the concept of Hierarchy of Effects, which divides attitude formation into three levels: (1) the standard learning level (cognition-affect-conation = attitudes based on cognitive information processing), (2) the low-intervention level (cognition-affect-conation = attitudes based on behavioral learning processes), (3) the experience level (affect-conation-cognition = attitudes based on experiential learning).

The CAC pattern is important for researchers to discover the relationships and formation of attitudes that can explain a variety of behaviors around us and predict future intentions and behavior. The successful development of the CAC pattern has led many researchers to use it to study and gain empirical confirmation of users’ attitudes towards information and communication systems. That is, to investigate the relationship between cognition, affect, and conation (Huang et al., 2019). This pattern has been used and validated in various communication fields including tourism, advertising, food marketing, and information delivery research (Lavidge and Steiner, 1961; Lv et al., 2022; Shu et al., 2022). Similarly, it is also relevant and adequate for understanding health knowledge communication. In terms of health spread, many scholars have used this model to study attitudes towards the use of health apps or willingness to vaccinate against COVID-19, etc. (Huang et al., 2019; Yu and Chen, 2021). Yu and Chen utilized CAC pattern to examine the association between media use, health beliefs, and vaccination intentions among Chinese citizens in the context of the new ecological pattern of communication (Yu and Chen, 2021). *Via* this study, cognition is derived from the perceptions and beliefs produced by the distribution of health knowledge in digital media, including the application of health knowledge to their circumstances. Affect relates to the public’s emotional response to the knowledge presented by comparing it to their own health knowledge. Conation indicates that the public takes appropriate actions, such as adopting knowledge of health communication or making purchases in response to marketing in the posts. This is a typical example to use this model in a health communication study, which gives us a theoretical foundation.

Many studies have explored the relationship among the three factors of attitude. The study by Hong et al. hypothesized that cognitive factors trigger consumers’ emotional responses, leading to behavioral change on the basis of the CAC pattern (Lu B. et al., 2022). They successfully confirmed this hypothesis by investigating the experiences and decision-making behaviors of MAR consumers. This study demonstrates that the CAC pattern can be used in the study of response sequences to learning. However, the order of affective, knowledge, and behavioral responses has not been addressed in the existing literature on health knowledge communication. In conclusion, we believe that the CAC pattern is appropriate and able to address how consumer learning and perceptions are translated into behavior in full alignment with our research objectives.

### 2.2. Hypothesis and research model

The media’s role in health knowledge communication is to gather knowledge that readers want to know about specific health topics and then integrate plan, and disseminate that knowledge (Yang, 2011; Wang, 2012; Ning, 2019; Yu, 2019). Readers, on the other hand, peruse the knowledge on digital media by the firms and compare it with their health issues. As a consequence, people create a new self-cognition of health and two behavioral goals applying the knowledge or acquiring the required items. This emotion is intricately tied to a person’s current emotional state. When it comes to acquiring knowledge, the cognition of the condition may be acquired from earlier objective aspects (such as medical reports) or the consumer’s subjective awareness. Therefore, we hypothesize in this paper that

*H1*: Consumer self-cognitions are positively correlated with their affect and conation.

*H1a*: Consumer self-cognitions are positively correlated with their affect.

*H1b*: Consumer self-cognitions are positively correlated with their conation.

In terms of reader access to information (i.e., knowledge sharing by companies), what may be most directly recognized is the correctness and authenticity of the knowledge. The quality standards for internet health information include Credibility, Content, Design, Disclosure, and Interactivity. Sub-criteria include Credibility and Content (Afful-Dadzie et al., 2021), which fall under the umbrella of each of the preceding criteria. It has been shown that the reader’s perception of the information’s quality (accuracy, authenticity, etc.) has a major impact on its effectiveness (DeLone and McLean, 2004; Jin et al., 2021). When the usefulness of knowledge fulfills expectations, favorable attitudes will result. People’s cognitions of information are primarily influenced by content, presentation, and provider. Hence, these three dimensions are selected as the main determinants influencing consumers’ attitudes to knowledge.

The knowledge content incorporates the user’s health knowledge as well as additional information about the product or service in issue. When this brief but deep knowledge is relevant to consumer preferences, it is more likely to elicit good assessments and influence the consumer’s subsequent choice. In terms of presentation, the current ecology of communication has switched from offline to online, which has a considerable influence on the shape of information. The dissemination of health knowledge has shifted from an offline forum to online graphics, Q&As, WOAs, etc., displayed in voice, video, live broadcasts, etc. It is a benefit (Huo et al., 2019) because the public may have more quick access to knowledge through digital media, which decreases the knowledge of communication errors and improves communication efficiency. Additionally, it is easier to grasp the product’s qualities and functions. Certain suppliers may have a significant influence on consumer decision-making (Chakraborty, 2019). There are now two major content development models on the market: one for health-related authority teams, such as media organizations or hospitals, and the other for key opinion leaders (KOL). Typically, the engagement of ‘authoritative experts’ who are well-liked and well-recognized by the public on digital media is required to increase the credibility of online content. Therefore, we hypothesize in this paper that

*H2*: People’s cognitions of content in health knowledge marketing are positively correlated with their affect and conation.

*H2a*: People’s cognitions of content in health knowledge marketing are positively correlated with their affect.

*H2b*: People’s cognitions of content in health knowledge marketing are positively correlated with their conation.

*H3*: People’s cognitions of presentation in health knowledge marketing are positively correlated with their affect and conation.

*H3a*: People’s cognitions of presentation in health knowledge marketing are positively correlated with their affect.

*H3b*: People’s cognitions of presentation in health knowledge marketing are positively correlated with their conation.

*H4*: People’s cognitions of provider in health knowledge marketing are positively correlated with their affect and conation.

*H4a*: People’s cognitions of providers in health knowledge marketing are positively correlated with their affect.

*H4b*: People’s cognitions of providers in health knowledge marketing are positively correlated with their conation.

**Figure 2:**
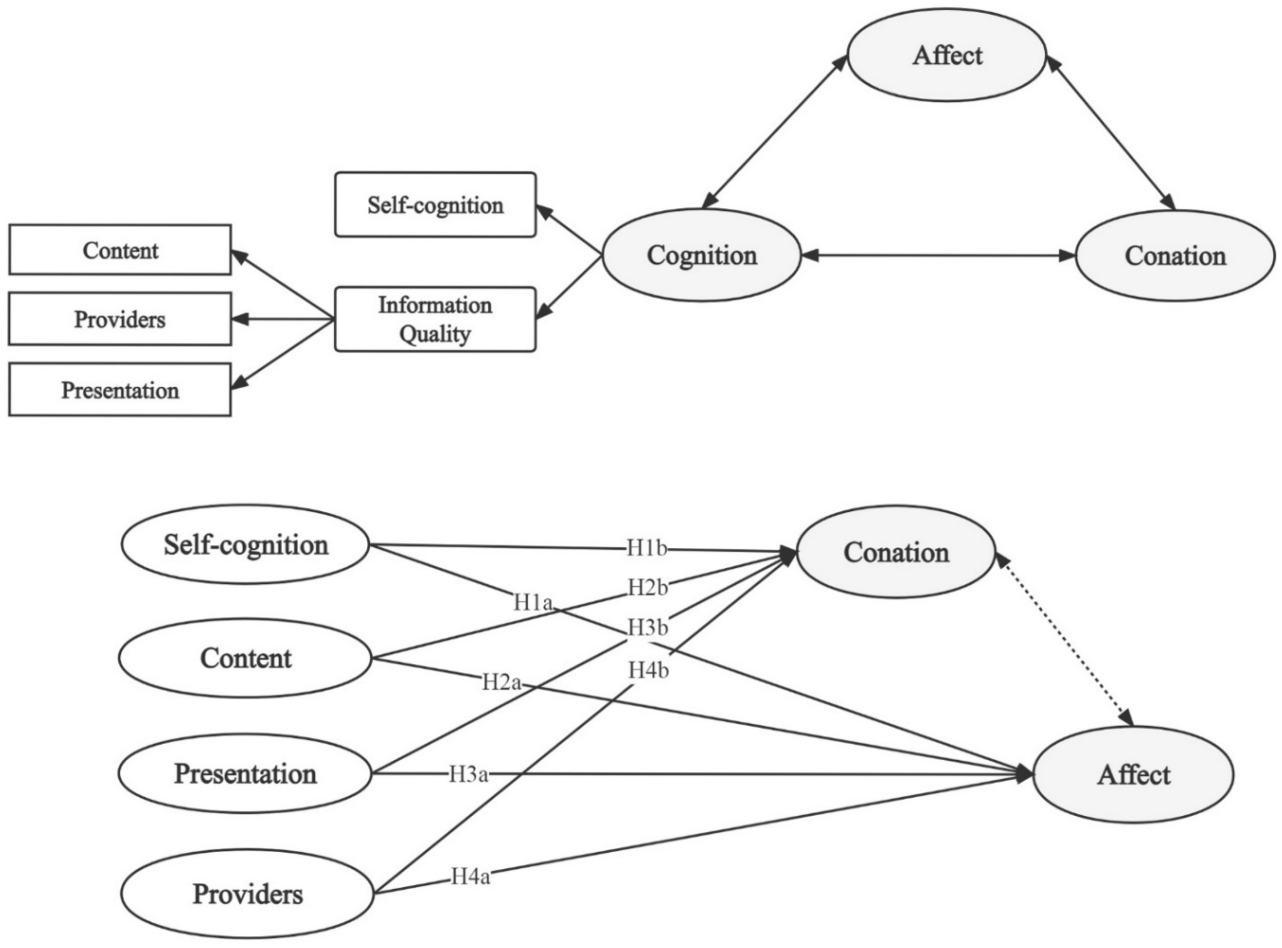
Research model.

The research model in this paper is shown in Figure 2.

## 3. Research design and methodology

### 3.1. Procedure and data overview

This study investigates the logical connection between users’ attitudes toward health knowledge marketing in digital media platforms and the elements that impact these views. Therefore, we surveyed digital media users to see whether they had acquired health knowledge on these platforms and if it has affected them. This work uses a questionnaire to conduct empirical research, using a convenience sample and snowball sample to disseminate a questionnaire to individuals in Guangzhou, China that have focused on ‘Dr. Dingxiang’. The questionnaire was also distributed on many platforms, including Weibo and WeChat, to collect a significant sample size for the study.

The questionnaire consists of three major components: introduction, investigating participants information, and the scale part. We informed the participants of the goal of the research and the principle of voluntary participation in the first section. In addition, the survey was performed anonymously, and a stringent data security and preservation plan was implemented to ensure that only members of the research team had access to the data. Section two of the survey asked about participants’ demographics, including their gender, age, and educational attainment. In the third section of this investigation, the research team covered 25 predictive variables for four latent variables. We included test questions in the survey to ensure the answers were accurate and quickly weed out any incorrect entries. Based on the results, the questions were amended, and the final questionnaire was disseminated.

### 3.2. Measure

Using well-established scales from earlier studies, all important study questions were adapted for digital media health communication. This research evaluated health-related self-cognitions, the cognitions of information quality, affect and conation. All of the questionnaire’s scales were measured on a Likert scale using a 5-point scale. Participants are required to choose a number from the scale.

For the purpose of gaging the participants’ self-perception of health, the scale was constructed based on their situation and health knowledge requirements (Brazier et al., 1992; Lin et al., 2009). Information Quality Scale is derived from (Laudon and Laudon, 2020; Afful-Dadzie et al., 2021), which includes three dimensions: information content, information presentation, and information provider. The affect component checks how consumers intuitively feel about this style of communication, while the conation part evaluates the user’s adoption of the knowledge and willingness to buy the product (Lavidge and Steiner, 1961; Moriarty, 1983; Breckler, 1984).

### 3.3. Sample

From 1 December 2021 to 30 December 2021, 335 questionnaires were collected. However, owing to the screening criteria of questionnaire test questions and short response times, 21 of them were considered invalid. On 314 legitimate surveys, we had a return rate of 93.73%, indicating that the findings are trustworthy. The final sample consisted of 314 inhabitants of Guangzhou, China, as shown in Table 1. The sample consisted of 58% females and 42% males between the ages of 18 and 24. (54.14%).

**Table 1:** Demographic profile of respondents (*N* = 314).

Measure	Category	*N*	Percent
Gender	Male	132	42.04
	Female	182	57.96
Age	<18	0	0
	18-24	170	54.14
	25-30	48	15.29
	31-40	50	15.92
	41-50	32	10.19
	51-60	12	3.82
	Over 61	2	0.64
Education	Primary school	8	2.54
	Junior High School	14	4.45
	Highschool	38	12.10
	College	60	19.11
	Undergraduate	184	58.59
	Postgraduate	10	3.18
	Male	132	42.04

### 3.4. Data analysis

For data analysis, this research used SPSS 23.0. The data analysis consisted of three primary components. Initially, the data were examined for internal consistency using Cronbach’s alpha (CA) and for the validity of the indicators using factor loading. Next, correlation analysis was undertaken on the latent variables (cognition, affect, and conation). The independent and dependent variables proposed in this research were subjected to concluding regression analysis.

## 4. Results

### 4.1. Validity and reliability

In this study, the internal consistency index was used to test the reliability of the scale. If the Cronbach’s alpha coefficient is between 0 and 1, then the larger the coefficient, the higher the internal consistency of the scale (Hair et al., 2019). The analysis revealed that the internal consistency reliability of Cronbach’s alpha coefficients was 0.565 for the self-health perception scale, 0.642 for the presentation scale, 0.665 for the content scale, 0.593 for the provider scale, 0.721 for the affective attitude scale, and 0.714 for the conation scale. The degree of consistency among all subscale items is satisfactory. The total internal consistency reliability of the scale was 0.903 (> 0.7), suggesting that the questionnaire is very reliable. Therefore, the questionnaire’s scales show good reliability.

Table 2 shows the KMO and Bartlett’s spherical tests. It reveals that the KMO measure is 0.876, which is larger than 0.7 (Kaiser and Rice, 1974), and that the approximate Chi-square value for Bartlett’s test of sphericity is 3039.181. With this result (*df* = 300, *p* = 0.000), the significance test at the 1% level of significance is passed. This demonstrates that the scales used in this research are appropriate for factor analysis.

**Table 2:** KMO and Bartlett’s test.

Kaiser-Meyer-Olkin measure of sampling adequacy		0.876
Bartlett’s test of sphericity	Approx. Chi-Square	3039.181
	*df*	300
	Sig.	0.000

KMO, Kaiser-Meyer-Olkin.

The results of the Pearson correlation coefficient analysis for the latent variables are shown in Table 3, and the results of the Pearson correlation coefficient analysis between the predictor variables and the latent variables are presented in Table 4. Combining the two tables reveals that the *p*-values of the significance levels between the three latent variables (cognition, affect and conation) are all less than 0.01, refuting the null hypothesis and confirming the presence of correlations. All correlation coefficients were substantially positive and more than 0.5 (Cohen, 1988; Rumpf, 2004), demonstrating a straightforward positive association between each variable. The relationships between the three latent elements and the four predictive variables of cognition (self-cognition, content, presentation and design) also demonstrated similar results. To some extent, all of the information presented here confirms hypotheses 1–4.

**Table 3:** Pearson correlation coefficient table for cognition, affect, and conation.

	Affect	Conation	Cognition
Affect	1		
Conation	0.724^**^	1	
Cognition	0.649^**^	0.706^**^	1

** Correlation is significant at the 0.01 level (2-tailed).

**Table 4:** Pearson’s correlation coefficient table for the predictive variables and the latent variables.

	Self-cognition	Presentation	Content	Provider
Affect	0.548^**^	0.542^**^	0.523^**^	0.508^**^
Conation	0.671^**^	0.567^**^	0.545^**^	0.504^**^
Cognition	0.850^**^	0.864^**^	0.780^**^	0.761^**^

** Correlation is significant at the 0.01 level (2-tailed).

### 4.2. Regression analysis

Based on the results of this preliminary analysis, we conducted a regression analysis for the data. Predictor variables included affect, conation and four predictive variables of cognition. Dependent variables were scores on the conation or affect. Because past research has indicated that consumers’ affective responses are brought on by cognitive factors and then result in behavioral adjustments, the cognitive and conation components enter the regression equation first. In the second step, four predictive variables of cognition, including self-cognition, content, presentation, and provider, were used to fit regression equations to affect and conation components, respectively. In the final step, the four predictive variables and the affective component were fitted with the conative component to explore the relationships.

Table 5 shows the regression analysis results of cognition, affect and conations. The results show that the R-square and the adjusted R-square are more significant than 0.6, indicating that the model fits well. It indicates that cognition (*β* = 0.551, *p* = 0.000), affect (*β* = 0.476, *p* = 0.000) were statistically significant predictor variables for conation and cognitive components have a greater impact on conation if viewed as a whole.

**Table 5:** Regression analysis of cognition, affect and conation.^a^

Model 1	Unstandardized coefficients		Standardized coefficients	*t*	Sig.	Collinearity statistics	
	*B*	Std. error	Beta			Tolerance	VIF
(Constant)	–0.207	0.178		-1.161	0.246		
Affect	0.476	0.048	0.458	9.988	0.000	0.579	1.726
Cognition	0.551	0.062	0.409	8.904	0.000	0.579	1.726
*R* Square	0.620						
Adjusted *R* square	0.618						
*F*	254.092						
Durbin–Watson	1.995						

^a^Dependent variable: Conation.

Using the same method, the results of the regression analysis of each predictive variable of cognition on the affect and conation were counted as shown in Tables 6 and 7. As can be seen from Table 6, the fit of each predictor variable to affect is greater than 0.3, while as can be seen from Table 7, the fit of all predictor variables to conation is greater than 0.5. In terms of coefficients, self-cognition (*β* = 0.475, *p* = 0.000), content (*β* = 0.138, *p* = 0.006), presentation (*β* = 0.208, *p* = 0.004), and provider (*β* = 0.119, *p* = 0.018) coefficients for conation were also higher than their coefficients for affect (SC = 0.233, Con. = 0.184, Pre. = 0.232, Pro. = 0.181). From this result, each component fits better with the conation. However, such results might be erroneous if third-party variables are taken into account. The team has reservations about this result and suspects that an affective component may play a mediating role. In any case, however, in terms of results, the hypothesis that H1a, H2a, H3a, and H4a is proved by Table 6, and Table 7 proves H1b, H2b, H3b, and H4b. Self-cognition significantly influences conation, and each variable has the same influence on information quality.

**Table 6:** Regression analysis of predictive variables and affect.^a^

Model 2	Unstandardized coefficients		Standardized coefficients	*t*	Sig.	Collinearity statistics	
	*B*	Std. Error	Beta			Tolerance	VIF
(Constant)	0.441	0.217		2.032	0.043		
Self-cognition	0.233	0.064	0.217	3.632	0.000	0.522	1.915
Presentation	0.232	0.075	0.188	3.084	0.002	0.502	1.991
Content	0.184	0.052	0.200	3.507	0.001	0.573	1.744
Provider	0.181	0.053	0.191	3.424	0.001	0.598	1.671
*R* Square	0.423						
Adjusted *R* square	0.415						
*F*	56.602						
Durbin–Watson	2.072						

^a^Dependent variable: Affect.

**Table 7:** Regression analysis of predictive variables and conation (a).^a^

Model 3	Unstandardized coefficients		Standardized Coefficients	*t*	Sig.	Collinearity Statistics	
	*B*	Std. Error	Beta			Tolerance	VIF
(Constant)	0.044	0.206		0.214	0.831		
Self-cognition	0.475	0.061	0.425	7.773	0.000	0.522	1.915
Presentation	0.208	0.072	0.162	2.906	0.004	0.502	1.991
Content	0.138	0.05	0.144	2.765	0.006	0.573	1.744
Provider	0.119	0.05	0.121	2.369	0.018	0.598	1.671
*R* Square	0.517						
Adjusted *R* Square	0.511						
*F*	82.671						
Durbin-Watson	2.037						

^a^Dependent variable: Conation.

### 4.3. Mediation model test

When considering the effect of the independent variable *X* on the dependent variable *Y*, *M* is said to be the mediating variable if *X* affects *Y* through the influence variable *M* (Wen et al., 2004; Wen and Ye, 2014). As previously stated, the research team had a presumption that the model contained a mediating effect during the research process and used the regression model to carry out a preliminary test. The results of regression analysis of affect, each variable of cognition, and conation are shown in Table 8. When the affect is involved, the effect of cognition decreases significantly. Thus, it is possible that affect is a mediating variable. Therefore, we began to test the mediation model. First, the mediating effects of affect in the relationship between cognition subcomponents and conation were examined separately using Model 4 (Model 4 is a simple mediation model) in the SPSS Macro compiled by Hayes (2012). Table 9 shows that self-cognition, content, presentation, and provider are significant for conation. When the mediating variables are added, the cognition sub-components are still significant for conation. In addition, from Table 10, the upper and lower limits of Bootstrap 95% confidence intervals for the direct effect of each subcomponent on the effect of the conation and the mediating effect of the affect do not contain 0. It suggests that cognition can not only directly influence conation, but can also have an impact through the mediation of affect.

**Table 8:** Regression analysis of predictive variables and conation (b).^a^

Model 4	Unstandardized coefficients		Standardized coefficients	*t*	Sig.	Collinearity statistics	
	*B*	Std. Error	Beta			Tolerance	VIF
(Constant)	–0.167	0.18		-0.927	0.354		
Affect	0.478	0.047	0.46	10.218	0.000	0.577	1.733
Self-cognition	0.363	0.054	0.325	6.727	0.000	0.501	1.996
Presentation	0.097	0.063	0.076	1.541	0.124	0.487	2.052
Content	0.05	0.044	0.052	1.133	0.258	0.551	1.814
Provider	0.033	0.044	0.033	0.733	0.464	0.576	1.735
*R* Square	0.639						
Adjusted *R* Square	0.633						
*F*	109.154						
Durbin-Watson	1.975						

^a^Dependent variable: Conation.

**Table 9:** Mediating model test of affect.

Independent Variable	Regression equation		Fit index		Coefficient Sig.	
	Result variable	Predictive variables	*R* ^2^	*F*	*B*	*t*
Self-cognition	Conation	Self-cognition	0.45	255.134	0.748	15.973^***^
	Affect	Self-cognition	0.3	133.751	0.588	11.565^***^
	Conation	Affect	0.631	266.048	0.529	12.362^***^
		Self-cognition			0.437	9.522^***^
Content	Conation	Content	0.297	131.775	0.52	11.479^***^
	Affect	Content	0.274	117.656	0.48	10.847^***^
	Conation	Affect	0.562	199.243	0.627	13.704^***^
		Content			0.218	5.197^***^
Presentation	Conation	Presentation	0.322	148.049	0.728	12.168^***^
	Affect	Presentation	0.294	129.851	0.67	11.395^***^
	Conation	Affect	0.567	203.593	0.612	13.269^***^
		Presentation			0.318	5.582^***^
Provider	Conation	Provider	0.254	106.469	0.497	10.318^***^
	Affect	Provider	0.258	108.619	0.482	10.422^***^
	Conation	Affect	0.549	189.118	0.655	14.243^***^
		Provider			0.182	4.167^***^

^***^p = 0.000.

**Table 10:** Total effect, direct effect and intermediate effect breakdown table.

Independent Variable		Effect	Boot SE	BootLLCI	BootULCI	Proportion of effect (%)
Self-cognition	Total effect	0.748	0.055	0.641	0.857	
	Direct effect	0.437	0.05	0.346	0.545	58
	Indirect effect(s)	0.311	0.042	0.232	0.398	42
Content	Total effect	0.52	0.055	0.408	0.625	
	Direct effect	0.218	0.044	0.14	0.312	42
	Indirect effect(s)	0.301	0.039	0.227	0.379	58
Presentation	Total effect	0.728	0.052	0.626	0.828	
	Direct effect	0.318	0.059	0.197	0.428	44
	Indirect effect(s)	0.41	0.057	0.303	0.525	56
Provider	Total effect	0.497	0.061	0.381	0.618	
	Direct effect	0.182	0.05	0.083	0.28	37
	Indirect effect(s)	0.315	0.048	0.222	0.412	63

The examination of direct and indirect effects reveals that the direct influence of self-cognition on conation is more substantial than the indirect effect of content *via* the affect. The mediation model test may essentially corroborate the claim that customer attitudes in health knowledge marketing correspond to the standard learning level (cognition-affect-conation). The pattern of attitude generation is generally consistent with existing research findings (Qin et al., 2021; Zhang et al., 2021), which Qin et al. concluded that “The results reveal that consumers’ cognitive evaluation of MAR applications stimulates their affective reactions, which eventually create conative behaviors.” in a study on mobile augmented reality. Therefore, the structural relationships between latent variables, standardized path coefficients, and hypothesis testing results are summarized in Table 11. All the assumptions have been tested (*p* < 0.05). The order of the four factors’ influence degree on affect is: self-cognition > content > provider > presentation. The order of influence degree on conation was: self-cognition > presentation > content > provider. Cognition can directly affect the conation through the mediation of the affect from the mediation model test. Moreover, the direct effect of self-cognition on the conation is more obvious. The indirect effect of content through affect is more significant than the direct effect.

**Table 11:** Results of hypothesis testing.

Hypothetically	Path	Std. Coeff	Conclusion	*p*
H1a: Consumer self-cognitions are positively correlated with their affect.	Affect ← self-cognation	0.217	Support	0.000^***^
H1b: Consumer self-cognitions are positively correlated with their conation.	Conation ← self-cognation	0.425	Support	0.000^***^
H2a: People’s cognitions of content in health knowledge marketing are positively correlated with their affect.	Affect ← content	0.200	Support	0.001^**^
H2b: People’s cognitions of content in health knowledge marketing are positively correlated with their conation.	Conation ← content	0.144	Support	0.006^**^
H3a: People’s cognitions of presentation in health knowledge marketing are positively correlated with their affect.	Affect ← presentation	0.188	Support	0.002^**^
H3b: People’s cognitions of presentation in health knowledge marketing are positively correlated with their conation.	Conation ← presentation	0.162	Support	0.004^**^
H4a: People’s cognitions of providers in health knowledge marketing are positively correlated with their affect.	Affect ← provider	0.191	Support	0.001^**^
H4b: People’s cognitions of providers in health knowledge marketing are positively correlated with their conation.	Conation ← provider	0.121	Support	0.018^*^

^***^p < 0.000, ^**^p < 0.01, ^*^p < 0.05.

## 5. Discussion

Through regression analysis and mediating effects analysis, this study initially developed a model of user’ attitudes towards health knowledge communication. CAC pattern was used as the theory for this study, guiding us to categorize and test attitudes into three components: cognition, affect and conation. The results confirm that users’ cognition and affect positively influence conation, and that affect acts as a mediating factor to help users form behavioral intentions based on their cognition of attitudes. Using the CAC model, our study fills a research gap regarding users’ perceptions of health knowledge communication. Based on the results, we present three findings that encompass normative content production, consumer focus, and improved consumer experience.

### 5.1. Regulating the content production

The results show that the formation of consumers’ attitudes in health knowledge marketing belongs to the standard learning level. The formation path is from cognition to affect to conation, based on the attitude from cognitive information processing. The implication is that we must pay attention to both consumer cognition and affect if we want to promote further the generation of consumer conation intention, which ignoring any of them will affect the conation intention directly. In this process of consumer conation, the formation of consumer attitude is not a ‘Decision-making’ hedonism, nor is it a low-intervention model like the purchase of necessities. Without the formation of underlying cognition, there is no further generation of affect and conation. In health knowledge dissemination, knowledge sharing is where consumers first encounter with knowledge and products, and where the first impressions are created. As one of the channels for companies to export their brand image, digital media platforms should check all links before transmission, including knowledge acquisition and organization. Knowledge itself should be the priority rather than product marketing. If the health knowledge cannot be transmitted to the readers or consumers accurately, it is hard to come up with subsequent affect and conation. Only by standardizing the content production mode and balancing the scales of knowledge and products can we bring greater effectiveness to consumers and more benefits to ourselves.

### 5.2. Focusing on the consumers

Consumer conation mostly comes from affect attitude and consumer self-cognition. In health knowledge marketing, readers or consumers’ self-cognition comes from the cognition of their health level of life, which is related to one’s knowledge level and the understanding of health knowledge. Emotional value primarily provides consumers with intangible benefits such as sensory and emotional attachment to satisfy their inner self-definition (Ajitha and Sivakumar, 2017). As a result, providing customers with relevant and interesting content can help them fulfill their own needs and create emotional value, which may ultimately influence their next course of action. The actions that readers or consumers take can be divided into two categories when they are exposed to health-related marketing: One is to implement healthy living measures, such as refuse eating High-Calorie foods and staying up late. The other is to buy recommended products. According to the results, people’s intention and determination to take actions to health live are firmer. More than 60 percent of the participants still choose to carry out the suggestions even though they know it is difficult. Regarding purchasing intention, consumers were more neutral having a desire to buy knowledge products recommended but would still hesitate to buy or not to buy. It also proves that consumers tend to be more rational about health products. The more rational consumers are, the more difficult to produce consumer conation. Therefore, enterprises and media should be act as consumer-centric, hearing the voices of consumers and trying to understand consumer needs. Using big data analytics to forecast popular demand. For example, in the live tape, the anchor team must make the selection and rehearse in advance. The first and foremost aspect of health knowledge marketing is understanding what kind of health needs the public has, what kind of health knowledge should be disseminated, and how to integrate health knowledge to gain more recognition from readers or consumers.

### 5.3. Enhancing consumer experience

The influence degree of each component of information quality on affect attitude is as follows: content > provider > presentation. In line with Content-driven thinking, the audience for health knowledge marketing will continue to focus more on the content quality of the information itself, followed by the presentation and provider. Of course, good content attracts the attention of the audience. In this research, nearly 50% of the respondents prefer humorous and novel content. In the 2022 Jiemian Health Forum held in Shanghai on November 16, 2022, Zhu Fan, from Ruijin Hospital, Shanghai Jiaotong University School of Medicine, mentioned that in the current society, audiences and consumers are less likely to accept health knowledge content that is too long or too specialized. As a result, the article must be easily understandable, direct, logical, and clear without tiring the reader out, while maintaining scientific accuracy and precision. With the current trend of extensive entertainment and fragmentation of society, fragmented content has gradually changed people’s consumption habits, making people more impetuous in the face of information. We have the resources to acquire knowledge, but we have been immersed in this entertainment culture for a long time without realizing it. However, the solution to ‘amusing ourselves to death’ is not to ban whole entertainment. We do not lock the health on digital media platforms. As mentioned above, the first step of health knowledge marketing is knowledge transmission; the second is product marketing. Dig deeper into the needs of consumers and try new ways to improve the consumer experience to increase the visibility of the content.

The credibility of the information is the factor that the audience will look for in information. In social exchange theory, there is an important influence of specific information providers on users’ choice intention. A study on user-knowledge payment platforms by Rao et al. also supports this (Rao et al., 2011). Furthermore, the source of information is the key. To break through the Berlin Wall from the audience to the consumer, we should improve the information reliability from the provider’s identity. Health knowledge marketing requires a professional presence in disseminating knowledge or selling products. Without authority, the mere adaptation from the current popular science information on the internet to re-dissemination, or simply talking about some of the literature and materials, is undoubtedly the people’s health as a joke. Only gaining the audience’s and consumers’ trust in the source can further enhance the effectiveness of health knowledge marketing content. The more authoritative the information source is, the more it can win the trust of consumers. Therefore, digital media platforms should grasp the psychology of consumers’ trust in authority and produce more specialized information content.

The media habit of the audience has changed from the initial text media to the way of video and Text Media Integration at present. With the development of digital technology, the visual culture represented is rising, changing the way of enterprise content production and audience content acceptance. In health knowledge marketing and dissemination, the Chinese audience’s exposure to health knowledge comes from digital platforms, such as WeChat and TikTok. Furthermore, they trust the content of video media more. It further shows that the media preference of the audience has changed significantly. Compared with the rational text content, the visual communication mode can convey the perceptual information more directly and gain the favor of audiences with its tangible and rational advantages. It is also the development path of health knowledge marketing in the future.

## 6. Conclusion

The results show that consumers’ attitudes are constructed through cognition, affect and conation in health knowledge marketing. The formation of consumer conation is linked to product cognition and affect. Therefore, the digital media of health knowledge should realize the balance between cognition and affect. Construct the bottom-level cognition and cultivate the emotional connection with users. Standardize the content production mode and create a good foundation for subsequent consumer behavior. Then focus on the internal information quality of digital media platforms, with diversified content production as the development direction. Information’s content, source, and mode of distribution have a diminishing effect on the emotional attitudes of consumers. The visualization and high-quality dissemination of health knowledge are beneficial in maintaining the stickiness of users of health knowledge on digital media and making users more willing to accept information from trusted platforms. Finally, operators should emphasize consumer portrait analysis, using digital media platforms user data to improve consumer group portraits. Aim at consumer demand by transferring the knowledge content related to them and promoting the generation of consumer purchase behavior. The multi-dimensional construction and optimization of consumers’ self-cognition, knowledge content, presentation, and providers in health knowledge marketing are carried out through digital media windows based on the level of attitude formation. Cultivate the emotional relationship between consumers and products to promote the generation of consumer will and behavior.

This study contributes to the expansion and improvement of findings about the influence of health knowledge marketing on users’ attitudes, hence enhancing our understanding of customers’ consumption behavior of health products *via* digital media. Companies and media involved in health knowledge marketing may use this research to enhance the efficiency and efficacy of health knowledge distribution and the purchase of health knowledge goods. It also encourages digital media platforms that communicate health knowledge to be improved continuously. By increasing the influence of health knowledge media platforms, we increase the channels for people to access health knowledge, promote health knowledge diffusion, improve citizens’ health literacy, and achieve equal conversation between the public and health care providers.

## Data availability statement

The original contributions presented in the study are included in the article/Supplementary material, further inquiries can be directed to the corresponding author.

## Author contributions

SZ and LZ: conceptualization, and writing—review and editing. SZ: formal analysis. SZ and XL: writing—original draft preparation. LZ: supervision, project administration, and funding acquisition. All authors contributed to the article and approved the submitted version.

## Funding

The research is supported by the General Projects of Guangdong Social Science Planning in 2021 (Grant No. GD21CSH07), and the National Natural Science Foundation of China in 2020 (Grant No. 71974189).

## Conflict of interest

The authors declare that the research was conducted in the absence of any commercial or financial relationships that could be construed as a potential conflict of interest.

## Publisher’s note

All claims expressed in this article are solely those of the authors and do not necessarily represent those of their affiliated organizations, or those of the publisher, the editors and the reviewers. Any product that may be evaluated in this article, or claim that may be made by its manufacturer, is not guaranteed or endorsed by the publisher.
